# The Effects of Tai Chi in Centrally Obese Adults with Depression Symptoms

**DOI:** 10.1155/2015/879712

**Published:** 2015-01-21

**Authors:** Xin Liu, Luis Vitetta, Karam Kostner, David Crompton, Gail Williams, Wendy J. Brown, Alan Lopez, Charlie C. Xue, Tian P. Oei, Gerard Byrne, Jennifer H. Martin, Harvey Whiteford

**Affiliations:** ^1^The University of Queensland School of Medicine, Brisbane, QLD 4102, Australia; ^2^Zhejiang Chinese Medical University, Hangzhou, Zhejiang 310053, China; ^3^Wuhan Sports University, Wuhan, Hubei 430079, China; ^4^Mater Health Services, Brisbane, QLD 4101, Australia; ^5^Metro South Addiction and Mental Health Services, Brisbane, QLD 4122, Australia; ^6^Centre for Neuroscience, Recovery and Mental Health, Brisbane, QLD 4122, Australia; ^7^The University of Queensland School of Public Health, Brisbane, QLD 4006, Australia; ^8^The University of Queensland School of Human Movement Studies, Brisbane, QLD 4072, Australia; ^9^Melbourne School of Population and Global Health, The University of Melbourne, Melbourne, VIC 3010, Australia; ^10^RMIT University School of Health Sciences, Melbourne, VIC 3083, Australia; ^11^The University of Queensland School of Psychology, Brisbane, QLD 4072, Australia; ^12^Mental Health Service, Royal Brisbane & Women's Hospital, Brisbane, QLD 4029, Australia; ^13^School of Medicine and Public Health, University of Newcastle, Callaghan, NSW 2308, Australia

## Abstract

This study examined the effects of Tai Chi, a low-impact mind-body movement therapy, on severity of depression, anxiety, and stress symptoms in centrally obese people with elevated depression symptoms. In total, 213 participants were randomized to a 24-week Tai Chi intervention program or a wait-list control group. Assessments were conducted at baseline and 12 and 24 weeks. Outcomes were severity of depression, anxiety, and stress symptoms, leg strength, central obesity, and other measures of metabolic symptom. There were statistically significant between-group differences in favor of the Tai Chi group in depression (mean difference = −5.6 units, *P* < 0.001), anxiety (−2.3 units, *P* < 0.01), and stress (−3.6 units, *P* < 0.001) symptom scores and leg strength (1.1 units, *P* < 0.001) at 12 weeks. These changes were further improved or maintained in the Tai Chi group relative to the control group during the second 12 weeks of follow-up. Tai Chi appears to be beneficial for reducing severity of depression, anxiety, and stress and leg strength in centrally obese people with depression symptoms. More studies with longer follow-up are needed to confirm the findings. This trial is registered with ACTRN12613000010796.

## 1. Introduction

Cardiovascular disease (CVD) is an increasingly prevalent health problem and has the highest consequential morbidity in the world. The association between depression and CVD has been identified and the increased risk due to this psychological factor is of similar order to the more conventional CHD risk factors such as smoking, dyslipidaemia, and hypertension [1]. In addition, the constellation of risk factors known as metabolic syndrome has also been shown to be highly predictive of the development of CVD [2, 3]. Metabolic syndrome is defined by the International Diabetes Federation as central obesity plus two of the following: elevated triglycerides, reduced HDL-cholesterol, elevated blood pressure, and/or elevated fasting blood glucose [4]. Central obesity itself, as a necessary requirement for defining metabolic syndrome, is a high risk factor for developing CVD [5] and is also associated with depression, which has been linked to an increased risk for CVD [6, 7]. Therefore, given the dramatically increased prevalence of CVD, control of depression and metabolic syndrome, especially in centrally obese adults with elevated depression symptoms, is of paramount importance to public health.

Previous studies have indicated the role of physical activity in depression and obesity control [8, 9]. However, many people, especially those who are aging, obese, or overweight, have increased physical limitations, which are perceived as a barrier to participation in more conventional physical activity such as gym-based exercise or aerobics due to fear of injuries or they are unable to or unwilling to participate in these exercises of training [10]. In addition, barriers of apathy, reduced motivation, and energy make physical activity challenging for people with depression [11]. Therefore, conventional types of physical activity may become more challenging for people with both depression and obesity. Thus, it is important to find a suitable exercise intervention for this population. Tai Chi, a form of low-impact exercise, may offer an alternative for this population.

Tai Chi is a form of mind-body movement therapy that has been practiced in traditional Chinese medicine for more than three hundred years. Although there are many different styles of Tai Chi, most consist of training of movement, breathing, and mind, with a strong focus on the mind, and share the low-impact nature. Tai Chi has been shown to have both physiological and psychological benefits [12]. This indicates that Tai Chi may be beneficial for reducing depression and helping to improve the sustainability of beneficial effects on measures of depression and metabolic syndrome which is important for CVD prevention and management. Moreover, three recent reviews have suggested that Tai Chi may have some beneficial effects on mental health in people with depression [13–15]. However, to date, no controlled studies have focused specifically on the effects of Tai Chi in centrally obese people with depression or depressive symptoms at risk of developing CVD.

The primary aim of this randomized controlled trial was therefore to determine the effects of Tai Chi on depression, as well as anxiety and stress in centrally obese adults with depression symptoms. In addition to depression symptoms, we included anxiety and stress as primary outcomes because the three are often coexistent and all of them are contributing factors in CVD [16, 17]. Secondary aims were to examine the effects on leg strength, central obesity, and other measures of metabolic syndrome. We also included leg strength because improvement in leg strength is important for this population to increase physical activity. We hypothesized that, after 12 and 24 weeks of Tai Chi intervention, there would be improvements in these measures in an instructor-led Tai Chi intervention group, relative to a usual medical care control group.

## 2. Methods

This study was approved by the Human Research Ethics Committees of the University of Queensland and the Princess Alexandra Hospital, Brisbane, Australia. Participants were recruited from local communities in Brisbane between October 2009 and March 2010, using a range of methods, including advertisements and referral from primary care physicians/general practitioners. Inclusion criteria were (1) age 18 to 80 years; (2) presence of central obesity defined by the International Diabetes Federation (waist circumference > 95 cm (men), 80 cm (women), and/or body mass index > 30 kg/m^2^) [4]; (3) general practitioner diagnosed depression; and (4) being on antidepressants for depression or having a short form Center for Epidemiologic Studies Depression Scale 10 (CES-D10) rating score of 10 or higher [18–20]. The CES-D10 shows good predictive accuracy when compared to the full-length 20-item version of the CES-D 20 (kappa = 0.97, *P* < 0.001) with a cut-off score for depression of 10 or more on the CES-D10 [19].

All potential participants attended screening assessment two to three months prior to baseline assessment (the “run-in period”). After baseline assessment, participants satisfying the inclusion criteria listed above were randomly allocated to either a Tai Chi intervention group or a wait-list usual medical care control group. In order to ensure intervention and control groups had similar distributions of major risk factors, all participants were initially stratified by age group (<40 years, 40–59 years, and ≥60 years), gender, and waist circumference (central obesity) (above or below gender-specific means) and whether or not the participant was on antidepressant medication for depression. Within each stratum, participants were then randomized to intervention or control group, using a computer-generated randomization schedule developed and conducted by an independent programmer. Intervention group participants attended 12 weeks of Tai Chi training, followed by 12 weeks of follow-up. The control group continued to receive usual medical care from their general practitioners. All participants provided written informed consent.

Intervention group participants attended an instructor-led group Tai Chi training program (3 sessions per week) during the 24 weeks of study under the guidance of an experienced Tai Chi instructor with more than 10 years of Tai Chi training. Participants also received a DVD demonstrating the Tai Chi exercise and were encouraged to practice the movements at home on days when they did not attend a group session. All participants continued their usual medical care throughout the study period. The exercises were adapted from the Kaimai style Tai Chi with low-impact nature as other styles of Tai Chi [21]. Each session lasted for 1 to 1.5 hours with 10-minute warm-up, 45-minute practice, and 10 to 25 minutes of cooldown. The session duration was progressively increased from 1 to 1.5 hours during the program.

The primary outcomes were severity of depression, anxiety, and stress symptoms assessed by the CES-D10 (CES-D) [19] and the Depression Anxiety Stress Scale 21 (DASS21) [22]. The CES-D is well known and remains as one of the most widely used instruments in the field of psychiatric epidemiology [20]. The DASS 21 is a well-validated measure of the severity of depression, anxiety, and stress [23]. Total score ranges from 0 to 42 for depression, anxiety, or stress, with higher scores indicating more severe depression, anxiety, or stress (depression: 0–9 = normal, mild depression = 10–13, moderate depression = 14–20, severe depression = 21–27, and extremely severe depression ≥ 28; anxiety: 0–7 = normal, mild anxiety = 8-9, moderate anxiety = 10–14, severe anxiety = 15–10, and extremely severe anxiety ≥ 20; stress: 0–14 = normal, mild stress = 15–18, moderate stress = 19–25, severe stress = 26–33, and extremely severe stress ≥ 34) [22].

Secondary outcomes included leg strength, central obesity (waist circumference), and other measures of metabolic syndrome defined by the International Diabetes Federation (including waist circumference, body mass index, blood pressure, fasting blood glucose, triglycerides, and HDL-cholesterol). Leg strength was assessed using a chair-stand test (number of stands completed in 30 seconds) (after the fasting blood measures test) [24]. Waist circumference was measured three times for each participant and results were recorded. The mean of the three measurements was taken as the final result. The waist circumference assessor stood in front of the participant to correctly locate the narrowing of the waist. The measurement was taken with an anthropometry measurement tape at the level of the narrowest point over the naked skin between the lower costal (rib) border and the iliac crest. When there was no obvious narrowing, the midpoint between these two landmarks was marked and the measurement was taken at the midpoint. Body mass index (BMI) was calculated using the formula BMI = weight (kg)/height (m)^2^. Body weight and height were measured three times for each participant and results were recorded. The mean of the three measurements was taken as the final result. Each participant rested for at least 5 minutes prior to blood pressure measurement in a chair with their back supported. Systolic blood pressure and diastolic blood pressure had been measured three times for each participant and results were recorded. The mean of the three measurements was taken as the final result. The blood samples were collected and assessed as per standard pathology procedures at the Princess Alexandra Hospital, Brisbane, Queensland, Australia.

Measurements were obtained at baseline and 12 and 24 weeks by a trained senior clinical nurse who was blinded to group allocation and findings from any previous assessments. All assessments were conducted by the same qualified personnel to minimize interassessor measurement error.

Physical activities (moderate, hard, and very hard activities) at baseline were assessed using a 7-day Physical Activity Recall Scale designed by Sallis et al. [25]. Retention and attendance at the Tai Chi group sessions and reasons for nonattendance were recorded by the Tai Chi instructor. Compliance with at-home practice recommendations and changes in other physical activities during the intervention were recorded using a physical activity diary.

Double data entry and verification were conducted by two independent research assistants. All analyses were performed using SAS or SPSS. Baseline characteristics for Tai Chi intervention group and control group were compared using *t*-tests for continuous variables and chi-square tests for categorical variables. The analyses were conducted based on intention-to-treat, with missing values imputed based on an assumption of no change. Mean changes and 95% confidence intervals (CI) were calculated for each outcome measure for changes from baseline to 12 and 24 weeks. Changes within each group were conducted by paired *t*-tests. Between-group differences in changes from baseline to 12 and 24 weeks were estimated (with 95% confidence intervals) and compared using generalized estimating equation models with time × group interactions and adjustment for baseline values.

The original sample size calculation was based on the mental health and metabolic findings (mean difference = 3 units and standard deviation of mean difference = 3 for depression; mean difference = 0.5 mmol/L and standard deviation of mean difference = 1 for fasting blood glucose) from a preliminary study, with a power of 80% and a two-sided significance level of 5%, resulting in at least 64 participants per group [26]. Assuming a dropout rate of 25%, we aimed at an initial recruitment target of 80 per group or 160 in total. It was anticipated that data from this number of participants would provide sufficient power to detect meaningful changes in all established measures of depression, anxiety, and stress symptoms, leg strength, central obesity, and other measures of metabolic syndrome based on the findings from the preliminary study [26]. Following an overwhelming response to the study recruitment, we increased the number of participants screened to 260, allowing increased power for post hoc subanalyses.

## 3. Results 

**Figure 1:**
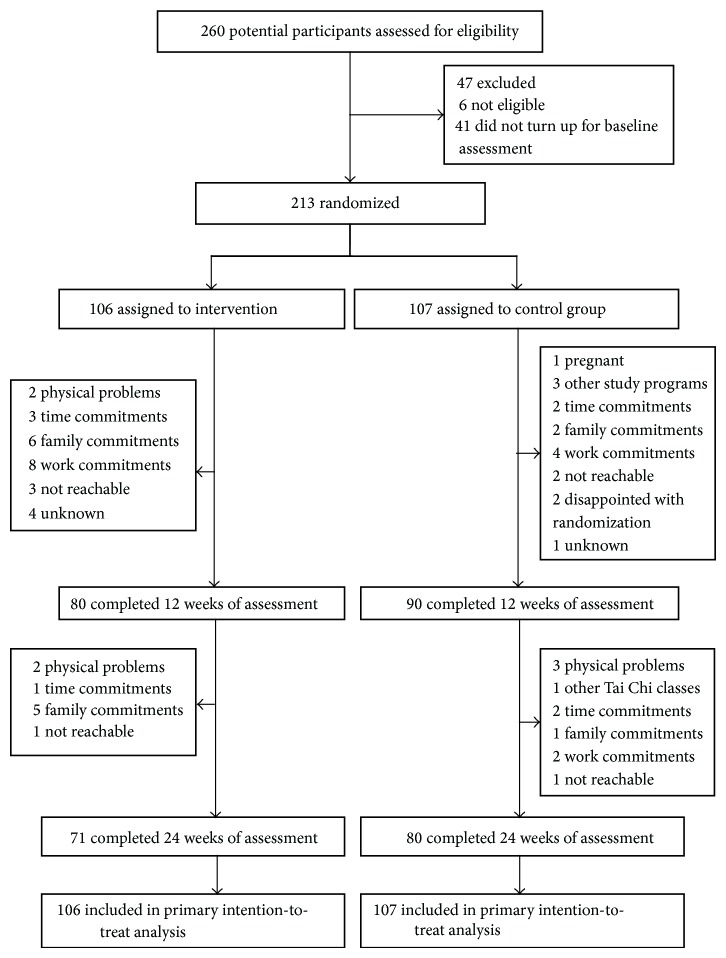
Flow of participants in the study.

Table 1 shows the baseline characteristics of the participants. Initially, 1,602 individuals responded to the study recruitment. We screened 536 respondents by telephone, of whom 290 were potentially eligible and were invited to attend the face-to-face screening assessment. In total, 260 respondents attended the screening assessment. At the end of the run-in period, 213 confirmed eligible participants (64 men and 149 women; age 19 to 77 years) were randomized to Tai Chi intervention (*N* = 106) or usual medical care control group (*N* = 107) (see Figure 1). The only statistical between-group difference at baseline was in depression symptoms assessed by CES-D10; the mean score was lower in the Tai Chi group (mean = 15.7) than in the control group (17.1) (*P* < 0.05). In terms of severity level of mental health, the participants had moderately severe levels of depression (DASS depression score = 19 units), anxiety (DASS anxiety score = 10 units), and stress symptoms (DASS stress score = 19 units) at baseline based on the defined categories of severity levels of depression, anxiety, and stress [20].

**Figure 2:**
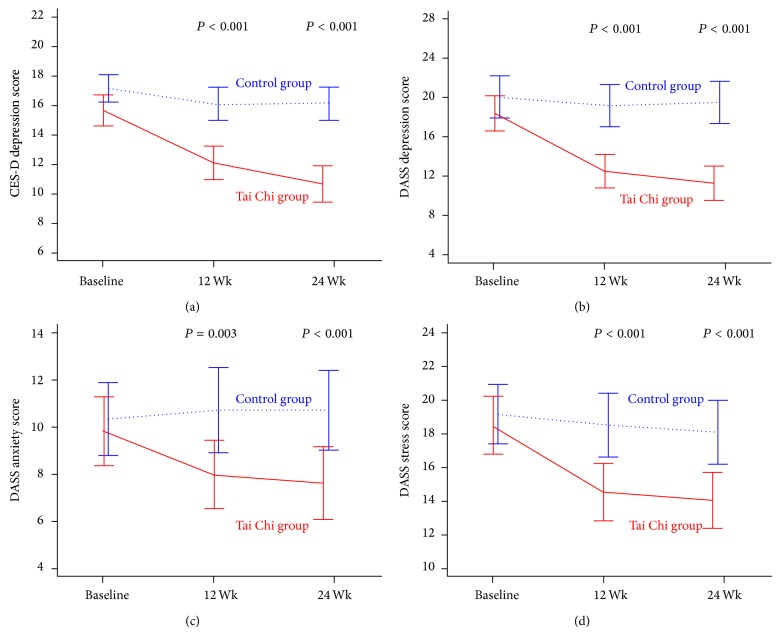
Mean changes in depression, anxiety, and stress scores at 12 and 24 weeks. The values shown are unadjusted means; I bars indicate 95% confidence intervals. *P* values assess difference in mean scores at 12 and 24 weeks, after adjusting for baseline. CES-D = Center for Epidemiologic Studies Depression Scale 10. DASS = Depression Anxiety Stress Scale 21.

Table 2 and Figure 2 show the changes in measures of depression, anxiety, stress, leg strength, waist circumference (central obesity), and other measures of metabolic syndrome during the intervention. At 12 weeks, there were statistically significant between-group differences in favor of the intervention group in CES-D depression (−3.0 units, *P* < 0.001), DASS depression (−5.6 units, *P* < 0.001), DASS anxiety (−2.3 units, *P* < 0.01), and DASS stress (−3.6 units, *P* < 0.001) symptoms scores and leg strength (1.1 units, *P* < 0.001). These changes were further improved or maintained in the intervention group relative to the control group during the second 12 weeks of follow-up, with significant between-group differences in favor of the intervention group in CES-D depression (−4.5 units, *P* < 0.001), DASS depression (−7.2 units, *P* < 0.001), DASS anxiety (−2.7 units, *P* < 0.001), and DASS stress (−3.6 units, *P* < 0.001) symptoms scores, as well as leg strength (1.6 units, *P* < 0.001). There were no changes in metabolic measures.

On average, Tai Chi group participants practiced the exercise program 4 sessions per week (2 sessions of supervised group sessions and 2 sessions at home) during the 24 weeks of study. At the end of the 24 weeks, 29% of participants had dropped out (see Figure 1). There were two adverse events during the study: one participant died due to heart failure and one was hospitalized with depression after expressing dissatisfaction with the randomization result.

**Figure 3:**
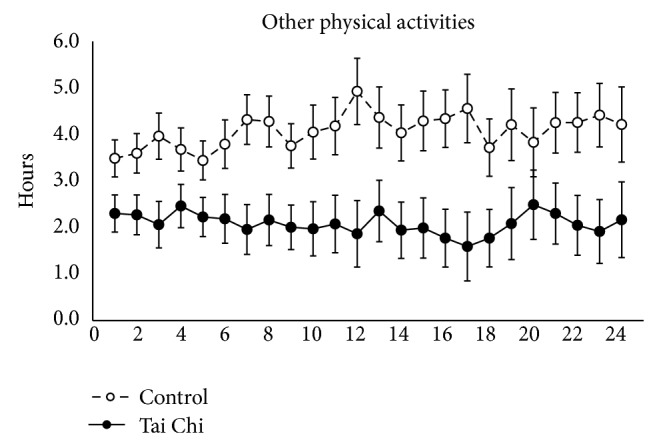
Mean changes in other physical activities during the 24 weeks of intervention (after start of Tai Chi training). I bars indicate standard errors.

In terms of total time spent on other physical activities, the control group participants spent more time than the Tai Chi group during the first 12 weeks and the second 12 weeks of follow-up (see Figure 3).

By 24 weeks after randomization, a few participants reported changes to their medication for depression control (6 of 106 intervention group participants versus 6 of 107 control group participants) and sleep (1 of 106 intervention group participants versus 0 of 107 control group participants), but no significant between-group differences were identified. There were no reported changes to medication for anxiety in either of the groups.

## 4. Discussion

This was the first study to examine the effects of Tai Chi in centrally obese adults with elevated depression symptoms, a specific and increasingly important population. The findings showed significant improvements in the severity levels of depression, anxiety, and stress symptoms, in the Tai Chi intervention group, relative to the usual care control group. In addition, there was also improvement in leg strength in the Tai Chi group at three months, compared with the control group. The changes in both mental health and leg strength were further improved or maintained in the Tai Chi intervention group during further three months of follow-up, compared with the control group.

The effects of Tai Chi on mental health in this community based sample of centrally obese adults with depression symptoms were consistent with those reported in previous studies of nonobese participants [13–15]. The change in depression symptoms in centrally obese adults with depression symptoms is important as this may help control obesity and ultimately eliminate the risk of obesity-related diseases such as CVD because there is strong relationship between depression and central obesity, suggested by Needham et al. [7]. Although the pathways linking Tai Chi with improvements in mental health are unclear, Tai Chi is a mind-body movement therapy that involves focusing the mind on movement or “energy flow” in a positive way in combination with gentle breathing and repeated movements; this may partially explain the mental health benefits of Tai Chi training. In addition, it may be possible that Tai Chi exercise could enhance central brain-derived neurotrophic factor activity, as has been shown with other forms of exercise [27, 28], which requires further study to confirm.

The improvements in leg strength are also consistent with findings from previous studies [26, 29]. As this challenging group of obese patients with elevated depression symptoms are usually less active and often suffer from low energy and physical limitations, improvement in leg strength may be a critical factor in increasing participation in physical activity more generally. Although Tai Chi involves gentle and slow movements, studies have now shown that it may improve leg strength without the high physical demand of conventional resistance training programs [26, 29], which may be partially explained by the fact that Tai Chi training consists of many leg movements.

In terms of other physical activities, the Tai Chi group spent less time than the control group during the intervention. As there was no between-group difference in physical activities at baseline, this indicates that the Tai Chi group might have reduced their previous physical activity time due to the time allocated to Tai Chi training during the study.

The finding of no significant changes in metabolic measures in this study may be explained by the fact that baseline glucose levels were not significantly elevated or that the intensity of the Tai Chi training was not high enough or that the improvements in metabolic factors may require changes in both activity and dietary measures and may take longer to achieve than changes in mental health and leg strength.

In this study, more than two-thirds of participants in both groups reported a range of chronic health problems (arthritis or rheumatism, diabetes, heart disease, hypertension, stroke, and lung disease). As no adverse events caused by the Tai Chi intervention were reported during the study, this suggests the safety of this gentle form of exercise for adults with multiple health challenges. In addition, although the dropout rate was relatively high, in line with most other exercise interventions, most participants who dropped out did so because of family and/or work commitments [26, 29].

The strengths of this study are that it was a community based randomized controlled trial and assessor blinded. One limitation is that we recruited participants with depression, identified by general practitioners in the primary care and being on antidepressants for depression and/or depression scores above a predetermined threshold. This may potentially limit the generalizability of the findings to a specific diagnostic category of depression, such as major depressive disorder. The lack of control for social contact and an alternative-treatment comparison group should be addressed in future work. In addition, we were unable to control for changes in medication, as all participants continued to receive their usual medical care during the study. Potentially, between-group contrasts may have been affected. However, whether or not the participant was on antidepressant medication for depression was initially stratified at randomization and the same number (only 6) of participants in each group changed their medication for depression during the study, so this did not affect comparisons.

## 5. Conclusions

This study has shown beneficial effects of a Tai Chi program on depression symptoms, as well as anxiety, stress, and leg strength in centrally obese participants with depression symptoms. Tai Chi appears to be a beneficial strategy to augment usual medical care in improving mental health and leg strength for these patients. More studies with long term follow-up are now required to confirm the findings and assess the sustainability of the benefits.

## Figures and Tables

**Table 1:** Demographic and clinical characteristics of the participants at baseline.

Characteristic	Tai Chi	Control
	(*N* = 106)	(*N* = 107)
	Mean (SD)	Mean (SD)
Age—years	52 (12)	53 (11)
Female sex—number of patients (%)	75 (70.8)	74 (69.2)
Higher school or higher education (number of patients) (%)	88 (83)	91 (85)
Employment—number of patients (%)		
Paid work	50 (47.2)	52 (48.6)
No paid work	17 (16.0)	24 (22.4)
Unable to work due to sickness or injury	15 (14.2)	16 (15.0)
Retired or other	24 (22.6)	15 (14.0)
Medication—number of patients (%)		
Depression	67 (63.2)	63 (58.9)
Anxiety	7 (6.6)	4 (3.7)
Sleep	8 (7.5)	11 (10.3)
Self-reported coexisting chronic conditions—number of patients (%)^a,b^		
0	36 (35.6)	38 (35.5)
1	32 (31.7)	37 (34.6)
≥2	33 (32.7)	32 (29.9)
CES-D depression score	15.7 (5.5)	17.1 (4.9)
DASS depression score	18.4 (9.4)	20.2 (11.4)
DASS anxiety score	9.8 (7.6)	10.3 (8.0)
DASS stress score	18.5 (8.8)	19.1 (9.2)
Leg strength test (number of stands in 30 seconds)	16.3 (5.2)	15.3 (4.3)
Waist circumference (cm)	105.5 (11.4)	105.8 (3.9)
Body mass index (kg/m^2^)	34.8 (6.6)	35.1 (7.1)
Fasting blood glucose (mmol/L)	5.9 (1.7)	6.1 (1.7)
Systolic blood pressure (mmHg)	122.8 (15.1)	123.8 (15.1)
Diastolic blood pressure (mmHg)	78.4 (8.2)	78.2 (8.3)
Triglycerides (mmol/L)	1.6 (1.5)	1.4 (0.8)
HDL-cholesterol (mmol/L)	1.1 (0.3)	1.2 (0.3)
Physical activities (hours)		
Moderate activities	4.3 (4.3)	5.3 (6.2)
Hard activities	0.5 (1.6)	0.5 (1.9)
Very hard activities	0.1 (0.6)	0.2 (0.7)

^a^Conditions included arthritis or rheumatism, diabetes, heart disease, hypertension, stroke, and lung disease; the number of conditions per participant ranged from 0 to 6.

^b^5 participants missing.

CES-D = Center for Epidemiologic Studies Depression Scale 10.

DASS = Depression Anxiety Stress Scale 21.

**Table 2:** Changes in primary and secondary outcomes and other physical activities during the 24 weeks of intervention (ITT analyses)^a^.

Variables	Comparison	Mean change from baseline (95% CI)		Between-group difference (95% CI)	
		Tai Chi (*N* = 106)	Control (*N* = 107)	Tai Chi versus control	*P* value^b^
CES-D depression score	3 mths versus baseline	−3.6 (−4.7 to −2.6)	−1.1 (−1.8 to −0.4)	−3.0 (−4.3 to −1.8)	**<0.001**
	6 mths versus baseline	−5.0 (−6.2 to −3.8)	−1.0 (−1.9 to −0.2)	−4.5 (−5.9 to −3.0)	**<0.001**

DASS depression score	12 wks versus baseline	−5.9 (−7.6 to −4.1)	−0.9 (−2.4 to 0.5)	−5.6 (−7.7 to −3.5)	**<0.001**
	24 wks versus baseline	−7.2 (−9.0 to −5.3)	−0.6 (−2.2 to 1.0)	−7.2 (−9.4 to −5.0)	**<0.001**

DASS anxiety score	12 wks versus baseline	−1.8 (−3.0 to −0.6)	0.4 (−0.7 to 1.4)	−2.3 (−3.8 to −0.8)	**0.003**
	24 wks versus baseline	−2.2 (−3.3 to −1.1)	0.4 (−0.6 to 1.4)	−2.7 (−4.1 to −1.2)	**<0.001**

DASS stress score	12 wks versus baseline	−4.0 (−5.6 to −2.4)	−0.6 (−2.0 to 0.7)	−3.6 (−5.5 to −1.6)	**<0.001**
	24 wks versus baseline	−4.4 (−6.2 to −2.7)	−1.1 (−2.3 to 0.2)	−3.6 (−5.5 to −1.6)	**<0.001**

Leg strength (number of stands in 30 s)	12 wks versus baseline	1.5 (1.0 to 2.1)	0.5 (0.0 to 0.9)	1.1 (0.4 to 1.8)	**0.001**
	24 wks versus baseline	2.0 (1.3 to 2.7)	0.5 (0.0 to 1.0)	1.6 (0.7 to 2.4)	**<0.001**

Waist circumference (cm)	12 wks versus baseline	−1.4 (−2.2 to −0.7)	−1.4 (−2.2 to −0.7)	−0.0 (−1.0 to 1.0)	0.97
	24 wks versus baseline	−1.2 (−2.1 to −0.3)	−1.8 (−2.7 to −0.9)	0.6 (−0.6 to 1.9)	0.32

Body mass index (kg/m^2^)	12 wks versus baseline	−0.1 (−0.2 to 0.1)	0.0 (−0.2 to 0.2)	−0.1 (−0.3 to 0.2)	0.54
	24 wks versus baseline	−0.1 (−0.3 to 0.1)	0.1 (−0.1 to 0.4)	−0.2 (−0.5 to 0.1)	0.17

Fasting blood glucose (mmol/L)	12 wks versus baseline	0.1 (−0.1 to 0.4)	−0.0 (−0.2 to 0.2)	0.1 (−0.2 to 0.4)	0.43
	24 wks versus baseline	0.1 (−0.1 to 0.3)	0.1 (−0.1 to 0.3)	−0.1 (−0.3 to 0.2)	0.62

Systolic blood pressure (mmHg)	12 wks versus baseline	1.2 (−0.3 to 2.7)	0.5 (−1.6 to 2.5)	0.5 (−1.7 to 2.7)	0.64
	24 wks versus baseline	1.0 (−0.3 to 2.4)	0.4 (−1.5 to 2.3)	0.4 (−1.7 to 2.5)	0.71

Diastolic blood pressure (mmHg)	12 wks versus baseline	0.7 (−0.3 to 1.6)	−0.2 (−1.3 to 0.8)	0.9 (−0.3 to 2.1)	0.15
	24 wks versus baseline	0.1 (−0.8 to 1.0)	−0.1 (−1.0 to 0.9)	0.2 (−1.0 to 1.4)	0.77

Triglyceride (mmol/L)	12 wks versus baseline	−0.06 (−0.24 to 0.12)	0.03 (−0.07 to 0.13)	0.01 (−0.15 to 0.16)	0.94
	24 wks versus baseline	−0.12 (−0.37 to 0.13)	−0.01 (−0.11 to 0.09)	−0.01 (−0.19 to 0.16)	0.88

HDL-cholesterol (mmol/L)	12 wks versus baseline	0.0 (0.0 to 0.1)	0.0 (−0.0 to 0.1)	0.0 (−0.0 to 0.1)	0.65
	24 wks versus baseline	0.1 (0.0 to 0.1)	0.1 (0.0 to 0.1)	−0.0 (−0.0 to 0.0)	0.93

Other physical activities (hours)	Total in first 12 wks	Mean = 25.6	Mean = 47.3	−21.9 (−36.7 to −7.2)	**0.004**
	Total in second 12 wks	Mean = 24.4	Mean = 50.5	−26.1 (−46.6 to −5.5)	**0.013**

^a^Values were calculated with intention-to-treat analyses.

^b^ *P* values were calculated with generalized estimating equations analyses after adjustment for baseline.

ITT = intention-to-treat analyses.

CI = confidence interval.

CES-D = Center for Epidemiologic Studies Depression Scale 10.

DASS = Depression Anxiety Stress Scale 21.
